# Concurrent Scrub Typhus, Dengue, and Leptospirosis: A Rare Triple Co‐Infection: A Case Report and Comprehensive Literature Review

**DOI:** 10.1002/ccr3.71933

**Published:** 2026-01-23

**Authors:** Sulav Kumar Jha, Bistrit Dahal, Anamika Adhikari, Kushal Singh Basnet, Shivaditya Singh, Manaswi Acharya

**Affiliations:** ^1^ Department of Internal Medicine Manipal College of Medical Sciences Pokhara Nepal; ^2^ B.P Koirala Institute of Health Sciences Dharan Nepal

**Keywords:** case report, dengue, leptospirosis, scrub typhus

## Abstract

In endemic areas, acute undifferentiated febrile illness has a wide differential with overlapping features that can lead to misdiagnosis. Although triple co‐infection with scrub typhus, dengue, and leptospirosis is extremely rare, high clinical suspicion and early detection are vital to avoid delayed treatment, multi‐organ dysfunction, and mortality.

## 1 Introduction

Acute undifferentiated febrile illness is a common clinical presentation in tropical and subtropical regions, particularly during the monsoon season. In South Asia, vector‐borne and zoonotic infections, such as scrub typhus, dengue, and leptospirosis, are significant causes of febrile illnesses [1]. These infections often present with nonspecific and overlapping clinical features, including high‐grade fever, myalgia, gastrointestinal symptoms, and respiratory complaints, which can make early and accurate diagnosis difficult [2].

Dengue, an arboviral illness spread by *Aedes aegypti* , is hyperendemic in tropical and subtropical regions [3]. Leptospirosis is a tropical zoonosis, usually acquired through occupational exposure to rodents or other animals [4], while scrub typhus (tsutsugamushi disease) is caused by *Orientia tsutsugamushi* and transmitted via chigger mites [5]. Although each infection alone causes significant morbidity, their concurrence increases diagnostic difficulty and disease severity.

Although triple co‐infection with scrub typhus, dengue, and leptospirosis is exceedingly uncommon, identifying it early is essential because any delay in diagnosis or targeted management may lead to severe and potentially fatal complications. We report a rare case in a Nepali farmer who developed this triple co‐infection and subsequently progressed to ARDS, septic shock, and ventilator‐associated pneumonia, yet recovered with timely antimicrobial therapy and intensive supportive care. This case, therefore, underscores the need to sustain a high index of suspicion for multiple concurrent infections when evaluating acute febrile illness in endemic settings. In addition, we describe the clinical course, discuss key diagnostic difficulties, and stress the broader public health value of improving clinician awareness during seasonal outbreaks.

## 2 Case History/Examinations

A 45‐year‐old male presented with a one‐week history of high‐grade fever, with documented temperatures up to 103.4°F, accompanied by generalized body ache and retro‐orbital pain. The illness was further characterized by cough, palpitations, unintentional weight loss, and loose stools persisting over the preceding 5 days. On further inquiry, the patient also reported exertional shortness of breath and episodes of hemoptysis but denied a history of vomiting, jaundice, and hematochezia. There was no previous history of chronic illness such as diabetes, hypertension, or tuberculosis. The patient also denied symptoms of chest pain, orthopnea, peripheral edema, or substance use, including alcohol and tobacco. Notably, there was no history of exposure to known toxins such as organophosphates, carbamates, or wild mushrooms. Furthermore, the patient had no known drug history, no history of drug allergies, and no recent travel to endemic areas.

The patient was a farmer by occupation and had been actively engaged in rice field work during the monsoon season in Nepal. On dietary inquiry, he reported consuming a mixed diet and primarily using untreated spring water as his drinking source. Family history was non‐contributory, with no similar illness noticed among close relatives.

On general examination, the patient appeared acutely ill‐looking, thinly built, and lethargic, but was responsive, with pupils bilaterally reactive to light. His recorded temperature was 103.4°F, blood pressure was slightly low, and oxygen saturation was 92% on room air, while other vital parameters remained stable. Additionally, no characteristic necrotic eschar was noted. No lymphadenopathy or peripheral edema was noted.

Systemic examination revealed bilateral vesicular breath sounds with localized crepitus in the right intrascapular area; no additional adventitious sounds were appreciated. Cardiovascular examinations demonstrated normal S1 and S2 without audible murmurs, and abdominal examination showed a soft, non‐tender abdomen with no organomegaly. Additionally, neurological assessment was grossly intact, with no focal deficits.

## 3 Differential Diagnosis/ Investigations

Initial laboratory evaluation revealed hemoglobin 11.4 g/dL, hematocrit of 33.1%, and leukocytosis with neutrophilia (81%) and lymphopenia (17%). Additionally, platelet count was markedly reduced to 21,000/μL, with progressive thrombocytopenia observed during hospitalization. Random blood glucose was normal, with liver function tests demonstrating markedly elevated aspartate aminotransferase (546 U/L) and alanine aminotransferase (156 U/L), with total protein 5.8 g/dL, albumin 3.2 g/dL, and alkaline phosphatase 138 U/L. Renal function test showed hyponatremia (131 mmol/L) and hypokalemia (3.0 mmol/L). Coagulation profile revealed prolongation of PT (16.3s; control 12.1s) with INR of 1.39. Urine analysis revealed proteinuria (2.1 g/dL) and microscopic hematuria (4‐6RBCs/hpf), without casts and crystals. Cardiac enzymes, including creatine kinase and troponin I, were within normal limits.

In view of the acute febrile illness with myalgia, cough, retro‐orbital pain, thrombocytopenia, and elevated transaminases, the initial differential diagnosis included:

1. Scrub Typhus—supported by occupational exposure to rice fields, the monsoon season.
2. Dengue fever—supported by high‐grade fever, retro‐orbital pain, and marked thrombocytopenia.
3. Leptospirosis—considered due to agricultural exposure to contaminated water, gastrointestinal symptoms, and renal involvement.
4. Community‐acquired pneumonia and pulmonary tuberculosis—in view of cough, hemoptysis, and infiltration in chest radiograph.
5. Viral hepatitis—considered given the significant elevation of transaminases.
6. Typhoid fever.

Serological testing for HIV, HBsAg, and HCV was negative. Rapid antigen testing for COVID‐19 was negative. GeneXpert for tuberculosis was sent, with the result pending at the time of reporting, which later came out to be negative. Dengue, scrub typhus, and leptospirosis were initially screened using immunochromatographic rapid assays. Scrub typhus testing demonstrated IgM positivity with IgG negativity, supporting an acute infection. Dengue diagnosis was supported by IgM positivity together with NS1 antigen positivity, consistent with early dengue. Leptospirosis was identified through IgM‐positive immunochromatographic testing. Because only a single serum sample could be obtained due to the patient's remote residence, paired sera for demonstrating a fourfold rise in antibody titers were not available, and confirmatory serological assays such as IFA, IgM ELISA, and MAT were not performed. To strengthen diagnostic certainty and minimize the possibility of cross‐reactivity inherent to serology, PCR assays for all three pathogens were performed, each of which yielded a positive result, providing molecular confirmation of active concurrent infection. Based on the clinical presentation and serological results, a working diagnosis of rare triple co‐infection of scrub typhus, dengue, and leptospirosis was established.

**FIGURE 1:**
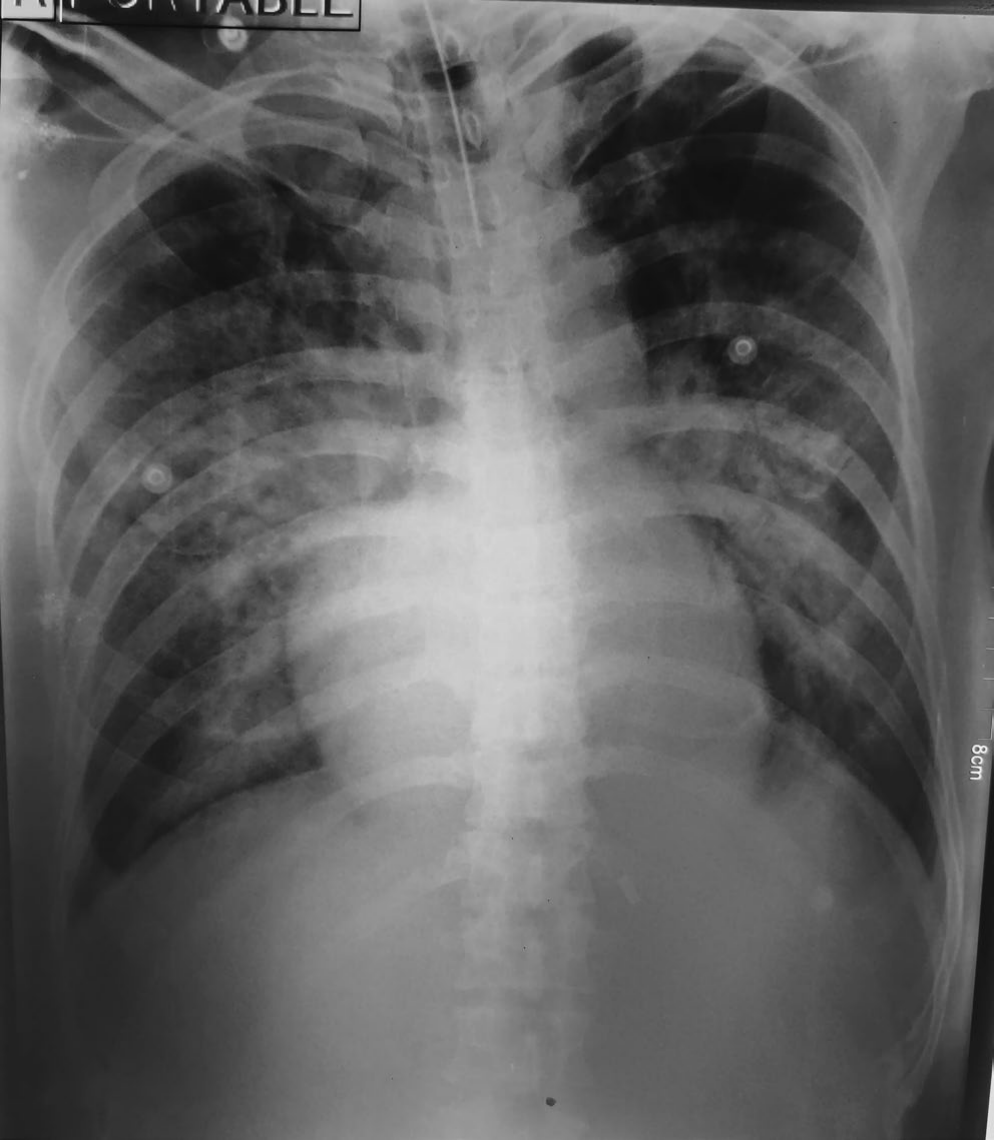
Chest X‐ray showing ARDS.

During hospitalization, the patient developed acute respiratory distress syndrome (ARDS) as shown in Figure 1 and septic shock, requiring mechanical ventilation. He subsequently developed ventilator‐associated pneumonia (VAP), with cultures growing *Acinetobacter* spp. To aid the comprehension of this complex clinical course involving triple co‐infection, a chronological timeline of key clinical events and interventions is provided in Table 1.

**TABLE 1:** Chronological timeline summarizing the patient's clinical course from symptom onset to discharge.

Day of illness	Clinical events	Interventions
Day 0 symptoms onset	Fever, headache, myalgia, retro‐orbital pain, cough, palpitations	Home care
Day 3	High‐grade fever, worsening fatigue, hemoptysis	Antipyretics from the local pharmacy
Day 6 (hospital admission)	Tachycardia, shortness of breath	Suspicion of AUFI, Screening using immunochromatography for dengue
Day 7	Worsening respiratory distress, persistent fever, and development of ARDS	ICU admission, Screening for leptospirosis, scrub‐typhus, malaria, Intubation and mechanical ventilation started
Day 8	Positive serologies for dengue, scrub typhus, and leptospirosis	PCR samples sent for confirmation
Day 9	Diagnosis confirmed‐ triple co‐infection‐ by serology, PCR, and clinical correlation	Targeted antimicrobials started
Day 11–13	Hemodynamic stabilization, improving oxygenation	Continued ventilatory support; improving hemodynamics
Day 15	Marked clinical improvement; transitioning off ventilatory support	Extubated, Shifted out of ICU
Day 17	Stable on ward, tolerating oral medication	Discharge process initiated

## 4 Treatment/Outcome/Follow‐Up

The patient was initiated on intravenous therapy with doxycycline 100 mg every 12 h alongside penicillin G sodium 1.5 million units every 6 h, along with supportive therapy including intravenous fluids, antipyretics, electrolyte correction, and mechanical ventilation for ARDS. Broad‐spectrum antibiotics were initiated for septic shock and tailored to VAP after culture results confirmed *Acinetobacte*r. Platelet transfusions were administered in the form of platelet‐rich plasma (PRP) to manage severe thrombocytopenia. A positive Coombs test (Rh+) was noted, indicating immune‐mediated hemolysis; red blood cell transfusions were not required, and hemoglobin levels were monitored closely.

Over the course of hospitalization, gradual clinical improvement was observed: fever resolved, platelet count increased, and liver and renal function tests were normalized. Mechanical ventilation was successfully weaned once ARDS resolved. The patient was discharged in a hemodynamically stable state, with improving laboratory parameters and complete clinical recovery confirmed at two‐week follow‐up.

## 5 Discussion

Acute undifferentiated febrile illness (AUFI) remains a major clinical challenge in tropical countries like Nepal, particularly during the monsoon season, when vector‐borne and zoonotic infections are highly prevalent [6]. Studies from Wangdi et al. reported that among cases of AUFI, viral etiologies were more common than bacterial etiologies, with dengue fever being the leading cause (11.8%), while bacterial infections such as leptospirosis (4.4%), typhoid (4.0%), and scrub typhus (4.0%) were also significant contributors, followed by influenza other than H1N1 (3.1%) [7]. Although dengue, scrub typhus, and leptospirosis account for a substantial proportion of AUFI cases and often share manifestations such as fever, myalgia, gastrointestinal disturbances, and hematological abnormalities, their overlapping and non‐specific presentations make early differentiation challenging [8, 9]. The occurrence of co‐infection with all three pathogens in a single patient is rare and poses additional diagnostic and therapeutic challenges, often necessitating a high index of suspicion and timely multidisciplinary management. Supporting this, a prospective study on thrombocytopenia‐associated febrile illness noted that among over 1000 patients, only two had triple co‐infections [10]. Another AUFI study from Himachal Pradesh (India) found triple co‐infection in just one patient of many studied, while dual infections were substantially more common [11].

Although dual co‐infections involving dengue with scrub typhus or leptospirosis are increasingly reported during monsoon AUFI outbreaks, published triple co‐infections remain extremely uncommon. In Nepal, Bhattarai et al. [6] reported dengue‐scrub typhus‐typhoid co‐infection during dengue outbreak, while reports from neighboring regions similarly describe dengue combined with scrub typhus and a third bacterial pathogen. To date, reports specifically involving the triad of scrub typhus, leptospirosis, and dengue are exceptionally rare, emphasizing the novelty of the current case. A comparative summary of previously reported cases is provided in Table 2 [12, 13, 14, 15, 16]. Also, clinically previously described triple co‐infections frequently exhibit severe thrombocytopenia and multi‐organ dysfunction, comparable to our patient's ARDS and shock, but differ in pathogen combination and exposure context. These findings illustrate how uncommon triple co‐infections are in published data and why they are underdiagnosed until more severe complications arise.

**TABLE 2:** Previously reported triple co‐infections involving tropical pathogens and their clinical outcomes (data extracted from published reports).

Author	Year	Country	Pathogens involved	Major complications reported	Outcome	References
Ahlawat P. et al.	2024	India	Malaria + Scrub typhus + Leptospirosis	Pancytopenia, AKI, Transaminits	Recovered	[12]
Sriram T. et al.	2025	India	*P. falciparum* + *P*. *vivax* + Scrub typhus + Dengue	Persistent fever, severe anemia, and thrombocytopenia	Recovered	[13]
Thawornkuno C. et al.	2020	Thailand	Dengue + Leptospirosis + Murine typhus	Fever, AKI, hepatic involvement	Recovered	[14]
Basyal S. et al.	2025	Nepal	Brucellosis + Scrub typhus + Typhoid	Hepatospleenomegaly, jaundice, thrombocytopenia	Recovered	[15]
Purkait S. et al.	2024	India	Dengue + Scrub typhus + Hepatitis E	Severe hepatitis, thrombocytopenia, and sepsis induced MODS	Fatal	[16]

Dengue, a mosquito‐borne arboviral disease caused by four DENV serotypes, poses a global health threat with outcomes ranging from mild fever to fatal shock, fueled by urbanization, travel, and weak control measures, with limited progress in prevention through emerging vaccines [17]. Like most viral illnesses, dengue is a typically self‐limiting disease, with most patients recovering uneventfully. This stage is referred to as dengue fever. In contrast, dengue hemorrhagic fever (DHF) represents the severe manifestation, marked by heightened vascular permeability that results in plasma leakage and a tendency for bleeding [3]. Clinically, dengue typically has clinical courses in three phases: febrile, critical, and recovery [3].

- Febrile phase: typically lasts for 3–7 days, with manifestations such as high temperature, retro‐orbital headache, arthralgia, anorexia, and backache. Also, from the second day of fever, there is leucopenia, thrombocytopenia, and rising hematocrit, accompanied by elevated hepatic transaminases such as ALT and AST.
- Critical phase: marked by transient vascular leak with rising hematocrit, hypoalbuminemia, and rise of shock, bleeding, and liver dysfunction.
- Recovery phase: clinically recognized by improvement in the well‐being of the patient.

A study by Chiao‐Hsuan Chao and group demonstrated that NS1 directly activates platelets via TLR4, promoting aggregation, adhesion to endothelial cells, and macrophage‐mediated phagocytosis of platelets, thereby causing thrombocytopenia [18]. Studies also have claimed that dengue reduces platelet production, increases platelet activation, and clearance [19]. Dengue has no proven antiviral; care is mainly supportive, and in severe cases, as in DHF, intensive care may be required, including fluid resuscitation, blood product support, and oxygen therapy. Recent computational studies have identified the cyclic peptide PYRRP as a promising dengue fusion inhibitor, demonstrating strong binding and stability with DENV envelope protein in molecular docking and dynamics simulation, highlighting its potential as a novel antiviral candidate [20].

Leptospirosis was first described in 1886 by Adolph Weil as a febrile illness characterized by jaundice, renal failure, splenomegaly, and conjunctival suffusion, particularly among individuals engaged in water‐related occupations [4]. Historical investigations later identified the causative spirochaete when Stimson in 1907 observed the organism in renal tissue and named it *Spirochaeta interrogans* due to its distinctive question‐mark morphology [21]. Today, leptospirosis remains a significant global zoonosis, with recent estimates indicating a substantial disease burden in populations exposed to livestock, rodents, inadequate sanitation, or recreational freshwater sources [22, 23].

Human leptospirosis presents with a wide spectrum of clinical features ranging from a mild, self‐limiting febrile illness to a rapidly progressive and potentially fatal condition with multi‐organ involvement, and patients typically exhibit non‐specific symptoms such as fever, chills, myalgia (often over the calves and lower back), and retro‐orbital headache, which overlap with other tropical infections [24]. These are referred to as anicteric leptospirosis, whereas icteric leptospirosis represents a more severe form of the disease with rapid deterioration and manifestations such as moderate elevation of liver transaminases, acute kidney injury, elevated serum amylase, transient thrombocytopenia, respiratory complications including pulmonary hemorrhages, and cardiac involvement with T‐wave abnormalities and myocarditis [4, 22, 24].

Normally, administration of oral doxycycline (500 mg twice a day for 7 days) is recommended, but amoxicillin (500 mg/day for 1 week to 10 days), ampicillin (500–750 mg/day for 1 week to 10 days), and azithromycin (500 mg/day for 3 days) can also be given for mild to moderate disease [25]. Patients with severe leptospirosis, which commonly manifests as renal and hepatic failure, are administered penicillin (penicillin G sodium at a dose of 1.5 million U/6 h) intravenously for a week [25].

Scrub typhus, a bacterial infection caused by arthropod‐borne gram‐negative obligate intracellular bacillus *Orientia tsutsugamushi*, transmitted through the bite of *Leptotrombidium* mite known as chiggers [26]. Although once considered geographically restricted to the “tsutsugamushi triangle,” studies suggest its expansion beyond traditional endemic zones, emphasizing the need for heightened suspicion even in atypical regions [27].

The clinical spectrum of scrub typhus ranges from mild self‐limiting fever to severe multi‐organ dysfunction involving the liver, kidneys, lungs, and central nervous system. The diagnosis is complicated by its nonspecific presentation, often mimicking other acute undifferentiated tropical fevers such as dengue, leptospirosis, and malaria [26]. A characteristic necrotic eschar, although considered pathognomonic, is absent in up to 50% of patients, depending on the skin tone and anatomical site of inoculation [28]. Also, eschar formation is rarely reported in the Southeast Asian region [29]. Thapa et al. also reported that only 6.5% of patients in their study developed an eschar [30]. The absence of characteristic necrotic eschar contributes to delayed diagnosis.

Hepatic dysfunction is commonly reported in scrub typhus due to disseminated vasculitis and immune‐mediated injury, and elevated transaminases are frequently observed [31]. Acute kidney injury may result from pre‐renal azotemia, vasculitis‐induced acute tubular necrosis, or direct renal involvement [32]. Furthermore, scrub typhus is one of the important causes of thrombocytopenia in tropical countries [10]. Respiratory involvement, ranging from mild pneumonitis to acute respiratory distress syndrome (ARDS), significantly increases mortality risk [33]. Historically, the cornerstone of treatment of scrub typhus is early initiation of doxycycline, which leads to rapid defervescence and improved outcomes [34]. Trials from Varghese et al. [35] concluded that combination therapy with intravenous doxycycline and azithromycin was a better therapeutic option for the treatment of severe scrub typhus than monotherapy with either drug alone.

In tropical co‐endemic settings, and particularly in cases of acute undifferentiated febrile illness, the serological diagnosis of dengue, scrub typhus, and leptospirosis remains challenging because there is considerable IgM cross‐reactivity among these infections. Although IgM‐based studies are widely used for early screening, multiple studies have shown that they often lack adequate specificity when these pathogens circulate together. For instance, dengue is known to trigger broad polyclonal B‐cell activation, and hence can generate false‐positive scrub typhus IgM results and non‐specific reactivity to Orientia antigens [36, 37]. Conversely, scrub typhus itself may also produce false dengue IgM positivity [38]. Likewise, diagnostic overlap is well described between dengue and leptospirosis, because dengue‐associated exaggerated IgM responses can lead to false‐positive Leptospira IgM ELISA [39]. In addition, cross‐reactivity between scrub typhus and leptospirosis has been attributed to similar mechanisms, including polyclonal activation and non‐specific IgM binding, and therefore misclassification is common unless confirmatory reference tests such as indirect immunofluorescence assay (IFA) or microscopic agglutination test (MAT) are performed [40]. Importantly, reports of triple IgM positivity across these diseases may more often represent assay cross‐reactivity rather than true co‐infection, especially during early illness or when antibody titres are low [36, 38, 39, 40]. Moreover, dengue infection may even cause false positivity in Leptospira MAT and in IgG/IgM‐specific immunofluorescence or immunoblot assays through cross‐reactions with leptospiral outer surface membrane proteins [39]. Collectively, the literature underscores that IgM serology alone cannot reliably discriminate between these infections, and molecular or reference assays are required for co‐infections.

This case demonstrates how concurrent infections may interact pathophysiologically. Dengue‐associated capillary leak and platelet activation [2, 41], scrub typhus‐related vasculitis [42], and leptospiral endothelial injury [43] acted synergistically, accelerating ARDS and septic shock. The overlap also creates diagnostic pitfalls: renal involvement and proteinuria may be misattributed to severe dengue alone [44], while thrombocytopenia and transaminitis can mask scrub typhus [45] or leptospirosis unless multi‐pathogen testing is pursued early in monsoon AUFI. Compared with previously reported triple co‐infections involving dengue and scrub typhus with other pathogens, our patient similarly developed profound thrombocytopenia and multi‐organ dysfunction but recovered fully with early dual bacterial coverage and intensive care, suggesting that rapid identification and treatment may offset the additive risk of co‐infection.

This case highlights the rare but clinically significant occurrence of simultaneous co‐infections with overlapping clinical manifestations such as fever, myalgia, headache, and conjunctival suffusion. Therefore, co‐infections complicate diagnosis due to overlapping non‐specific clinical features and laboratory abnormalities. With this case report, we would also like to highlight the rarity of triple co‐infection of dengue, scrub typhus, and leptospirosis. Early recognition, a high index of clinical suspicion even in non‐endemic areas, and prompt initiation of appropriate antimicrobial and supportive therapy were crucial in preventing severe complications and ensuring a favorable outcome. Notably, we present clinical features, diagnostic challenges, and the importance of serological testing in definitive diagnosis. Furthermore, we emphasize that delayed or missed diagnoses can lead to severe complications, including multi‐organ failure and septic shock, and effective management requires a high index of clinical suspicion and prompt diagnostic testing. Ultimately, clinicians practicing in endemic regions should remain vigilant for possible co‐infections in patients presenting with acute undifferentiated febrile illness, as timely intervention can significantly reduce morbidity and mortality.

## 6 Conclusion

Triple co‐infection with dengue, leptospirosis, and scrub typhus, although rare, should be actively considered in acute undifferentiated febrile illness in co‐endemic regions. This case highlights the need for strong clinical vigilance and the early use of confirmatory diagnostic testing to avoid misclassification due to serological cross‐reactivity. Prompt, pathogen‐directed therapy with supportive care can ensure favorable outcomes. In addition, integrated vector and zoonotic control strategies remain essential to reduce the overlapping risk of these infections in endemic settings.

## Author Contributions

**Sulav Kumar Jha:** conceptualization, data curation, supervision, writing – original draft. **Bistrit Dahal:** conceptualization, writing – review and editing. **Anamika Adhikari:** writing – review and editing. **Kushal Singh Basnet:** writing – review and editing. **Shivaditya Singh:** writing – review and editing. **Manaswi Acharya:** writing – review and editing.

## Funding

The authors have nothing to report.

## Consent

Written informed consent was obtained from the patient for publication of this report and accompanying images in accordance with the journal's patient consent policy.

## Conflicts of Interest

The authors declare no conflicts of interest.

## Data Availability

Data will be provided by the corresponding author upon reasonable request.
